# MicroRNA-425-5p Inhibits Lung Cancer Cell Growth *in Vitro* and *in Vivo* by Downregulating TFIIB-Related Factor 2

**DOI:** 10.1177/1533033819901115

**Published:** 2020-01-22

**Authors:** Xi Yu, Hong Zheng, Rongfei Sun, Xuejiao Qian, Ping Jiang, Bo Yang, Jiangbo Liu, Xiaoping Li

**Affiliations:** 1 Department of Respiratory, Tianjin First Central Hospital, Tianjin, China; 2 Department of Thoracic Surgery, Tianjin First Central Hospital, Tianjin, China

**Keywords:** microRNA-425-5p, lung cancer, cell growth, TFIIB-related factor 2, biomarker

## Abstract

Lung cancer is the most common cancer type with increasingly high incidence. MicroRNAs provide the potential biomarkers for lung cancer treatment. Thus, we aimed to investigate the function of microRNA-425-5p in lung cancer development and the underlying mechanisms. MicroRNA-425-5p overexpression inhibited A549 lung cancer cell proliferation *in vitro* and *in vivo*. On the other hand, microRNA-425-5p inhibition increased A549 proliferation. Mechanistically, the underlying mechanism by which microRNA-425-5p inhibits lung cancer cell growth was mediated through its ability in targeting and downregulating the TFIIB-related factor 2. Our results for the first time identified microRNA-425-5p as a tumor suppressor in lung cancer. Thus, microRNA-425-5p may serve as a potential therapeutic target for lung cancer.

## Introduction

Lung cancer is a severe and common disease in the worldwide.^1^ It is classified histopathologically as non-small cell lung cancer (NSCLC) and small-cell lung cancer. Non-small cell lung cancer accounts for about 75% to 80% of all lung cancer cases. Although lots of great achievements have been made, the survival rate of NSCLC remains still low.^2^ Specifically, the lack of specific biomarkers for early diagnosis and targeted therapy largely contribute to the poor outcomes for patients with lung cancer.^3^

MicroRNAs are involved in regulating the carcinogenesis of many cancer types and provide the potential biomarkers and tools for cancer treatment.^4,5^ For lung cancer, several studies have shown that certain microRNAs are correlated with characteristics of lung cancer subtypes.^6^ Thus, microRNAs might serve as useful molecular targets for personalized therapeutic strategies. Among them, miR-425-5p was reported to be abnormally expressed in various human cancers. Moreover, miR-425-5p was deeply associated with the developmental process of these cancer types.^7-12^ However, the role of microRNA-425-5p in lung cancer metastasis and the underlying mechanisms remain to be elucidated.

TFIIIB-related factor 2 (BRF2) is a subunit of TFIIIB complex, which plays critical roles in promoting tumor progression and/or metastasis. Moreover, BRF2 expression is markedly increased in gastric cancer, renal cancer, and melanoma. Recently, BRF2 was reported to be associated with poor prognosis of patients with NSCLC through promoting tumor epithelial–mesenchymal transition.^13^ Also, BRF2 was identified as a novel lineage–specific oncogene in lung squamous cell carcinoma.^14^ However, it is still not clear whether BRF2 can interact with other factors, which have more critical functions in patients with NSCLC. Therefore, it is essential to further investigate the factors that regulate BRF2 expression and function in the development of NSCLC.

In this study, we uncovered the expression and function of microRNA-425-5p in patients with NSCLC and NSCLC cell line A549 and identified miR-425-5p as a novel NSCLC suppressor. Bioinformatic analysis and luciferase reporter assay showed that BRF2 was a direct target gene of miR-425-5p. In summary, our work suggests that microRNA-425-5p may be a novel target for the clinical treatment of lung cancer.

## Materials and Method

### Non-Small Cell Lung Cancer Samples

Tumor tissue samples were collected from patients with NSCLC in Tianjin First Central Hospital. All experiments involved in human specimens were approved by the Ethics Committee of Tianjin First Central Hospital (Approve no.CEA20171125LC22).

### Cell Lines and Transfection

A549 lung cancer cells were cultured in Dulbecco Modified Eagle Medium (Thermo Fisher, Shanghai, China) with 10% fetal bovine serum (Sijiqing, Hangzhou, China), maintained in a humidified incubator (eg, at 37°C, 5% CO_2_). Negative control mimic (NC mimic), miR-425-5p mimic, negative control inhibitor (NC inhibitor), and miR-425-5p inhibitor were purchased from GenePharma (Shanghai, China). BRF2 plasmid and negative control plasmid (NC vector) were also synthesized from GenePharma. Lipofectamine 2000 (Invitrogen, Shanghai, China) was used for A549 cell transfection following the manufacturer’s instructions.

### Quantitative Real-Time Polymerase Chain Reaction

The quality of complementary DNA from the small number of cells was analyzed by real-time PCR. Polymerase chain reaction was performed using an AB7500 with 96-well plates as follows: first, 95°C for 10 minutes, followed by 40 cycles of 95°C for 15 seconds and 60°C for 1 minute. The primer sequences used in this study were: BRF2: Forward: TGGGTGCTGCGTCTTAATCAC, Reverse: AGGAGCTTCACTATCTGCATGT; GAPDH: Forward: CTGGGCTACACTGAGCACC, Reverse: AAGTGGTCGTTGAGGGCAATG; U6: Forward: GCTTCGGCAGCACATATACTAAAAT, Reverse: CGCTTCACGAATTTGCGTGTCAT; miR-425-5p: Forward: TGCGGAATGACACGATCACTCCCG, Reverse: CCAGTGCAGGGTCCGAGGT.

### Cell Counting Kit-8 Assay

Cell counting kit-8 (CCK-8) was used to examine the proliferative activity of A549 cell line. A total of 100 μL of cell suspension (5000 cells/well) was placed in a 96-well plate. This plate was preincubated for 24 hours in a humidified incubator (eg, at 37°C, 5% CO_2_). A total of 10 μL of CCK-8 solution was then added to each well of the plate, and the plate was incubated for 1 hour under the same conditions as described above. Finally, the absorbance at 450 nm was measured using an automatic microplate reader.

### Western Blot

Equal amounts of protein were resolved by 10% sodium dodecyl sulfate–polyacrylamide gel electrophoresis and transferred to polyvinylidene membrane (Bio-Rad, Shanghai, China). After blocking in 3% bovine serum albumin in phosphate-buffered saline with 0.05% Tween-20, the membranes were incubated with rabbit polyclonal anti-BRF2 antibody (1:1000; ab154658; Abcam, Shanghai, China) and rabbit monoclonal anti-glyceraldehyde-3-phosphate dehydrogenase antibody (1:5000; ab181602; Abcam). The secondary antibodies are horseradish peroxidase conjugated. The blots were visualized with chemiluminescence.

### Colony Formation Assay

A number of 1000 cells were plated into 6-well plates and then cultured for 2 weeks to allow colony formation. Colonies were stained with 0.1% crystal violet (Beyotime Biotechnology, Shanghai, China) in 50% methanol and 10% glacial acetic acid for counting.

### Luciferase Reporter Assays

Reporter plasmid pmirGLO Oct4 was co-transfected with NC mimic, miR-425-5p mimic, NC inhibitor, and miR-425-5p inhibitor into A549 cells. The pRL TK Renilla luciferase reporter vector was used as an internal control. Firefly and Renilla luciferase activities were measured by Dual Luciferase Reporter Assay system. All the results were expressed as firefly luciferase activity normalized to Renilla luciferase activity.

### Statistical Analysis

Statistical analyses were performed with the Statistical Package for Social Sciences 20.0 (SPSS, Chicago, Illinois). The continuous variables were assessed with the independent Student *t* test. All data are shown as mean ± standard deviation from 3 independent experiments. *P* < .05 was considered to be statistically significant.

## Results

### MicroRNA-425-5p Inhibits Lung Cancer Cell Growth

**Figure 1.**
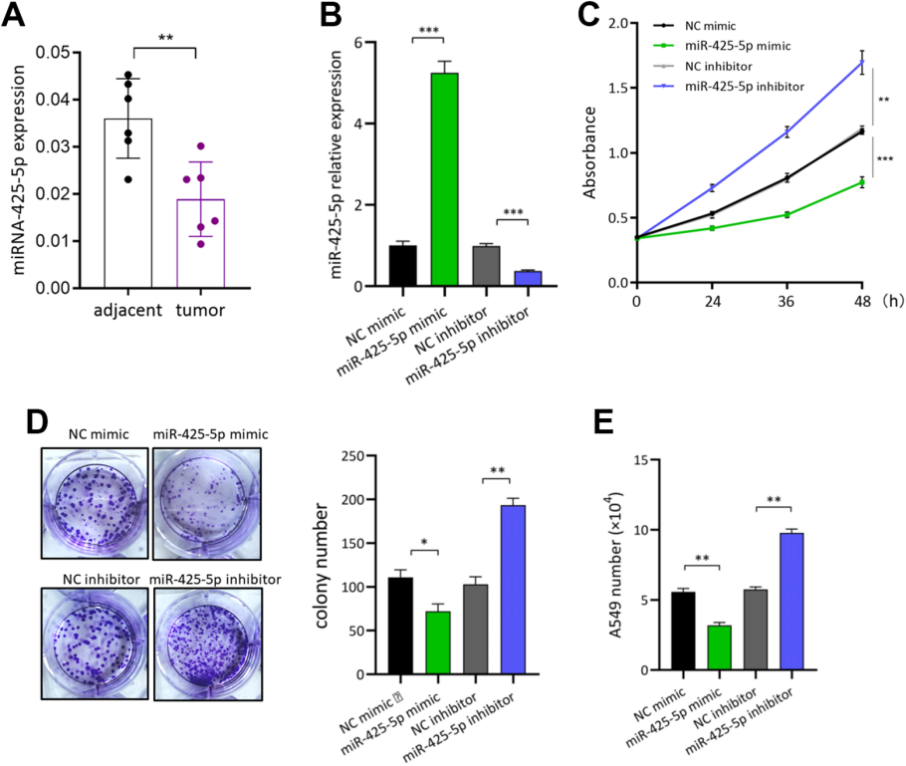
MicroRNA-425-5p inhibits lung cancer cell growth. A, microRNA-425-5p expression in NSCLC tumor tissues and tumor-adjacent normal tissues was detected by qRT-PCR. B, The overexpressed or downregulation of microRNA-425-5p in lung cancer A549 cell and detected by qRT-PCR (C) cell proliferation, (D) colony formation (E) cell numbers counting. NC mimic: negative control mimic. *P < .05, **P < .01, ***P < .001. NC indicates negative control; NSCLC, non-small cell lung cancer; qRT-PCR, quantitative real-time polymerase chain reaction.

First, we evaluated miR-425-5p expression in patients with NSCLC. Quantitative real-time polymerase chain reaction (qRT-PCR) results showed that the level of miR-425-5p was significantly decreased in NSCLC tumor tissues compared with that in the paired tumor-adjacent tissues (Figure 1A). To investigate the effect of miR-425-5p on lung cancer cell growth, miR-425-5p mimic and inhibitor were transfected into A549 cells NSCLC cell line. Quantitative real-time polymerase chain reaction results showed that miR-425-5p mimic or miR-425-5p inhibitor successfully overexpressed or suppressed miR-425-5p expression in A549 cells, respectively (Figure 1B and C). Importantly, the proliferation of A549 cells transfected with miR-425-5p mimic was significantly decreased when compared with those transfected control mimic, whereas miR-425-5p inhibitor increased A549 proliferation (Figure 1C). To further evaluate the effects of miR-425-5p on lung cancer cell growth, we performed the colony formation assay and found that miR-425-5p overexpression had a negative effect both on A549 cell clone formation capacity and cell number (Figure 1D and E). These results indicate that microRNA-425-5p inhibits lung cancer cell growth *in vitro*.

### Overexpression of miR-425-5p Inhibits the *in Vivo* Growth of Lung Cancer Cells

**Figure 2.**
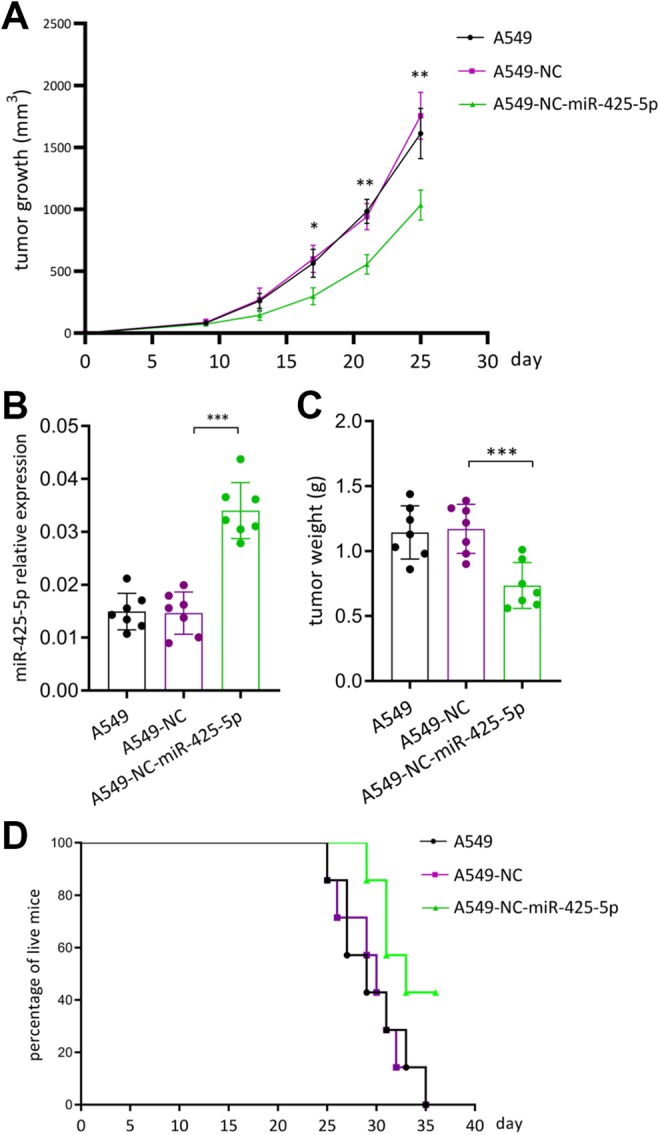
Overexpression of miR-425-5p inhibited tumor growth. A, A549 cells were inoculated to 6 to 10 weeks of nude mice and tumor volume measured by vernier caliper; (B) expression of 425 was detected by qPCR (C) tumor weighing (D) survival rates of mice. *P < .05, **P < .01, ***P < .001. qPCR indicates quantitative polymerase chain reaction.

Next, we established xenograft lung tumor models to detect whether miR-425-5p could also inhibit tumor growth *in vivo*. To this end, negative control mimics or miR-425-5p were stably transfected into A549 cells, then inoculated them subcutaneously into nude mice. As shown in Figure 2A, mice inoculated with A549 cells transfected with miR-425-5p developed smaller tumor size than both A549 group and A549-NC group (Figure 2A). After 25 days, mice were executed and miR-425-5p expression was detected by qRT-PCR. We found that mice inoculated with A549 cells transfected with miR-425-5p had the highest miR-425-5p expression and the lowest tumor weight than the other 2 groups (Figure 2C and D), suggesting that miR-425-5p also had a negative effect on tumor growth *in vivo*. On day 35, all mice inoculated with A549 cells and A549-NC cells died. In contrast, approximately 40% of mice inoculated with A549-miR-425-5p survived (Figure 2D). These results provided evidence that miR-425-5p is a tumor suppressor in mouse lung cancer model.

### TFIIIB-Related Factor 2 Is a Direct Target Gene of miR-425-5p

**Figure 3.**
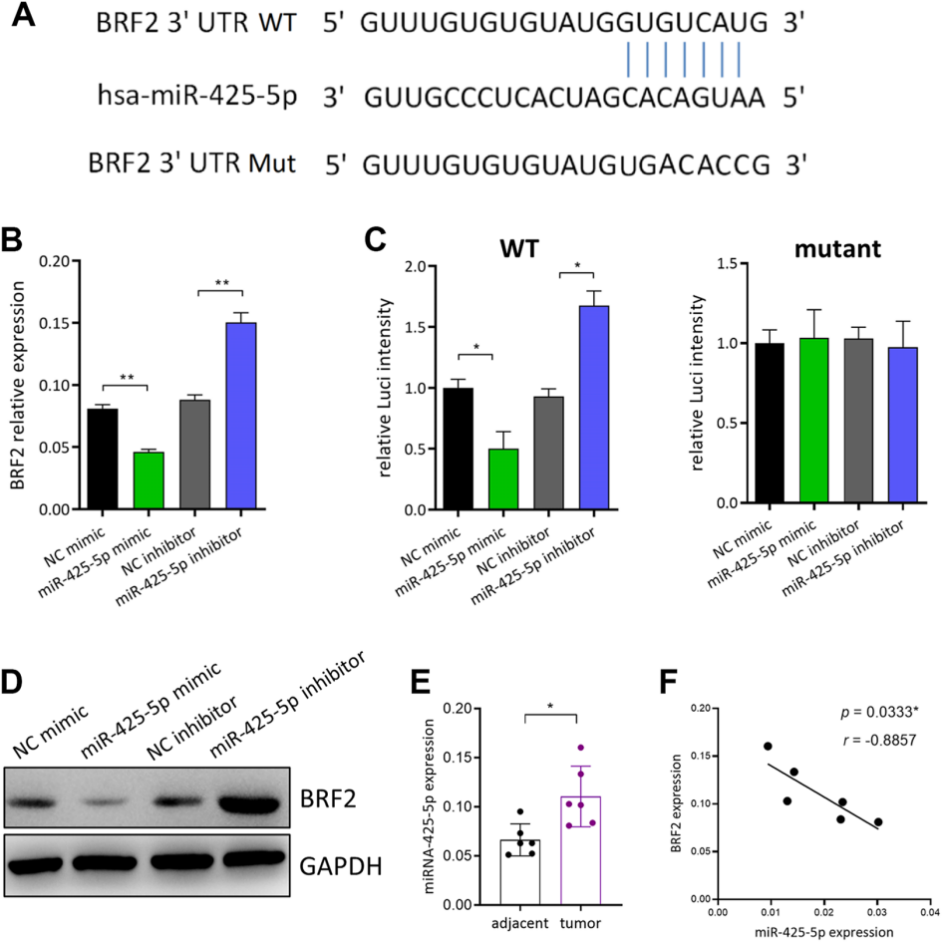
BRF2 is a direct target gene of miR-425-5p. A, TargetScan software was used to predict a binding site for miR-425-5p in the 3′-UTR of BRF2. B, Overexpression or downregulation of miR-425-5p in A459 cell and detect the expression of BRF2. C, Luciferase activity of a dual-luciferase reporter detecting BRF2 intensity when miR-425-5p overexpression or downregulation. D, Interactions between miR-425-5p and BRF2 *in vitro*. E, BRF2 expression in NSCLC tumor tissues and tumor-adjacent normal tissues was detected by qRT-PCR. F, The correlation between BRF2 mRNA level and miR-425-5p mRNA level was evaluated by Spearman rank correlation test. *P < .05, **P < .01, ***P < .001. BRF2 indicates TFIIIB-related factor 2; qRT-PCR, quantitative real-time polymerase chain reaction; 3′-UTR, 3′-untranslated region.

Since we had found that miR-425-5p suppressed lung cancer cell growth, we next looked at the mechanisms of action of miR-425-5p. Putative targets of miR-425-5p were examined by TargetScan bioinformatics algorithm. We identified 3′-untranslated region (3′-UTR) of BRF2 might be a target of miR-425-5p (Figure 3A). To further verify whether miR-425-5p suppressed tumor growth via targeting BRF2, miR-425-5p mimic or miR-425-5p inhibitor was transfected into A549 cells and then BRF2 expression was examined. As shown in Figure 3B, miR-425-5p significantly downregulated the mRNA expression of BRF2. Luciferase reporter assay was performed to confirm whether miR-425-5p directly regulated BRF2 expression through interacting with the predicted binding site. The results showed that the luciferase intensity in A549 cells transfected with miR-425-5p was significantly decreased as compared to that in control A549 cells. On the other hand, miR-425-5p overexpression or inhibition failed to alter the luciferase intensity in A549 cells transfected with mutant BRF2 3′-UTR (Figure 3C), suggesting that BRF2 was a direct target of miR-425-5p. These findings were further confirmed by the result that miR-425-5p decreased BRF2 protein level in A549 cells (Figure 3D). Moreover, we found increased BRF2 expression in NSCLC tumor tissues compared with that in the paired tumor-adjacent tissues (Figure 3E). Importantly, BRF2 expression showed strong negative correlation with miR-425-5p expression in NSCLC tumor tissues (Figure 3F). Collectively, BRF2 was a direct downstream target of miR-425-5p, and BRF2 expression was downregulated by miR-425-5p.

### MiR-425-5p Inhibits Lung Cancer Cell Growth by Downregulating BRF2

**Figure 4.**
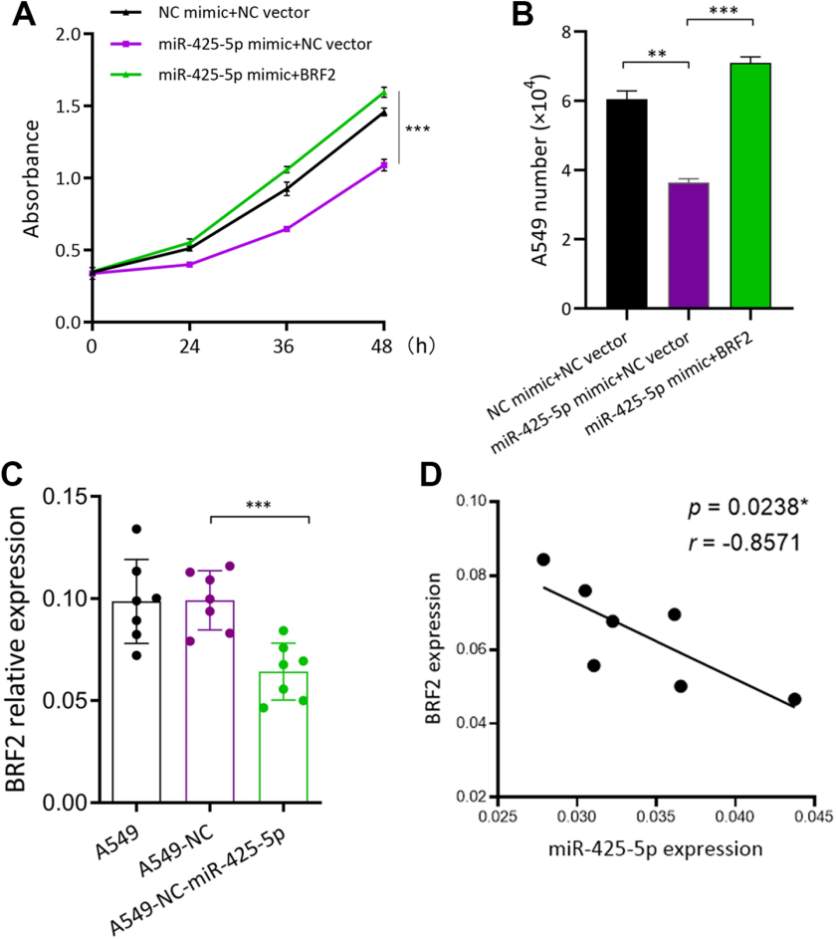
MiR-425-5p inhibits lung cancer cell growth by downregulating BRF2 (A) Overexpression miR-425-5p in A549 cells could inhibit cell growth but reversed by BRF2. B, Cell counting (C) BRF2 expression was detected by qPCR in tumor cells inoculated mice (D) Spearman rank correlation test; there was a significant negative correlation between miR-425-5p and BRF2 expression in tumor tissues. *P < .05, **P < .01, ***P < .001. BRF2 indicates TFIIIB-related factor 2; NSCLC, non-small cell lung cancer; qPCR, quantitative polymerase chain reaction.

We have proved that miR-425-5p downregulated BRF2 expression and also miR-425-5p inhibits lung cancer cell growth. Whether targeting of BRF2 is a potential mechanism of miR-425-5p affecting the lung cancer cell growth? To address this question, we transfected miR-425-5p mimic and BRF2 overexpression vector into A549 cells, in order to evaluate the role of BRF2 on the effect of miR-425-5p on A549 cells. The results showed that the inhibited A549 cell growth by miR-425-5p was rescued by BRF2 overexpression, as evidenced by CCK-8 assay and cell counting (Figure 4A and B). Moreover, in mouse tumors, the expression of BRF2 was markedly decreased in miR-425-5p tumors than that in A549 and A549-NC tumors (Figure 4C), indicating that miR-425-5p also negatively regulated BRF2 expression *in vivo*. Especially, Spearman rank correlation test further proved the significantly negative correlation between miR-425-5p expression and BRF2 expression in mouse tumor tissues (Figure 4D). In summary, our results indicate that miR-425-5p inhibits the growth of A549 cells both *in vitro* and *in vivo* through suppressing BRF2 expression.

## Discussion

Lung cancer, which is the most common primary lung malignancy, ranks the top both in the incidence and mortality rate of various malignant tumors worldwide.^1,15^ However, lots of abnormal gene expressions are involved in the occurrence and development of lung cancer, and there is no effective diagnosis method in the early stage, resulting in low survival rates of patients. According to cancer statistics, less than 15% of patients with NSCLC can be survived.^16^ Therefore, it is important to uncover the molecular mechanism of carcinogenesis of NSCLC subtype and to further explore the effective drug targets and diagnostic methods.

To our knowledge, malignant tumors are often caused by the imbalance between oncogenes and tumor suppressor genes in normal cells and abnormal expression and dysfunction of automatic regulation of normal genes. Until now, not only coding genes but also noncoding RNAs have been found to be involved in cancer metastasis.^17,18^ In molecular oncology, microRNAs play important roles in the occurrence and development of tumors both as proto-oncogene and tumor suppressor gene. So far, microRNAs have been extensively demonstrated to play regulatory roles in various cancer types, such as miR-9, miR-134, miR199, miR-425, and so on. They are involved in the development of cancer by regulating cell proliferation, apoptosis, invasion, and metastasis. However, the innate mechanism is still ambiguity.

Since Takamizawa *et al* first reported that the expression of let-7 was changeable in cancers, specifically in lung cancer, the opinion that microRNA profiles in malignant tumors may be related to the recovery of patients having lung cancers.^19^ MiR-21 shows significantly higher expression in lung hyperplasia atypical hyperplasia invasive carcinoma, metastatic carcinoma, and squamous cell carcinoma, and overexpression or downregulation of miR-21 in lung cancer cell H2170 caused that cell proliferation differs remarkably.^20^ Another study in Kaplan-Meier analysis showed that average survival rate of patients with higher expression of miR-150 is 40.8%, while 69.2% in the miR-150 low expression group, suggesting that high expression of miR-150 is associated with poor prognosis of patients.^21^ In this study, we first proved the biological functions of miR-425-5p in A549 lung cancer cell line and showed that overexpression of miR-425-5p could inhibit A549 lung cancer cell growth and cancer colony formation *in vitro*. Furthermore, through hypodermic inoculation of A549 cells with miR-425-5p overexpression, we demonstrated that miR-425-5p also had a negative effect on tumor growth *in vivo*.

Using the prediction algorithm TargetScan, we found that 3′-UTR of BRF2 contains a complementary binding site of miR-425-5p. BRF2 is a subunit of the transcription factor TFIIIB and involved in the production of small RNA catalyzed by RNA pol III.^22^ The relationship between BRF2 gene and TFIIIB determines its important role in tumorigenesis and development. In recent years, Lockwood *et al* found overexpression of BRF2 caused squamous cell carcinoma tumorigenesis,^14^ which presents a novel mechanism of lung squamous cell carcinoma tumor and also proves that BRF2 may be a specific gene of lung cancer. Furthermore, study on expression of BRF2 in patients with esophageal squamous cell cancer (ESCC) proved that higher expression of BRF2 was prevalent in ESCC, which have a relationship with deeper tumor invasion and microvessel density.^23^ Although BRF2 has been identified as the key protein in cancer development, the clear mechanisms that how it effects are still waiting to find out. In our study, we firstly showed the interaction of miR-425-5p and BRF2 in the NSCLC cancer type. Then, we further deciphered that miR-425-5p may inhibit lung cancer cell growth and tumor growth by targeting and downregulating BRF2, which was identified as a new mechanism of microRNA inhibit lung cancer.

Taken together, we showed that miR-425-5p could inhibit A549 lung cancer cell growth and proliferation and may function as a lung cancer suppressor by downregulating BRF2 expression, which provides a potential novel target and approach for lung cancer therapy in the future clinical cancer treatment.

## Abbreviations

BRF2: TFIIB-related factor 2
CCK-8: cell counting kit-8
ESCC: esophageal squamous cell cancer
NC: negative control
NSCLC: non-small cell lung cancer
qRT-PCR: quantitative real-time PCR
3′-UTR: 3′-untranslated region
